# Coxsackievirus B3 Cleaves INTS10 Through 3C Protease to Facilitate Its Replication

**DOI:** 10.3390/ijms27020996

**Published:** 2026-01-19

**Authors:** Luna Yuan, Liling Lin, Chunyan Bi, Xiaoyu Niu, Yang Chen, Yanru Fei, Guangtian Wang, Hui Wang, Yan Wang, Wenran Zhao, Zhaohua Zhong, Lexun Lin

**Affiliations:** 1 Department of Microbiology, School of Basic Medical Sciences, Harbin Medical University, Harbin 150081, China; yuanluna2022@163.com (L.Y.); nxy702809@163.com (X.N.); yanru_fei@163.com (Y.F.); wangyan@hrbmu.edu.cn (Y.W.); 2 Teaching Center of Pathogenic Biology, School of Basic Medical Sciences, Harbin Medical University, Harbin 150081, China; cy_hmu@126.com (Y.C.); wangguangtian0451@163.com (G.W.); wanghui727@163.com (H.W.); 3 Department of Cell Biology, School of Basic Medical Sciences, Harbin Medical University, Harbin 150081, China; zhaowr@hrbmu.edu.cn

**Keywords:** Coxsackievirus B, INTS10, snRNA processing, virus replication

## Abstract

Coxsackieviruses possess two proteases that are engaged in cleaving viral polyprotein and hijacking host cell processes such as RNA biosynthesis. Integrator subunit 10 (INTS10), a subunit of the integrator complex, facilitates the processing of small nuclear RNAs (*U1* and *U2* snRNAs) to regulate cellular transcription. We found that INST10 can be cleaved by Coxsackievirus B (CVB). Hence, we hypothesized that INST10 may play a role in CVB infection. In this study, INTS10 is identified as the substrate of CVB3 protease 3C (3C^pro^). The cleavage occurs at the residue Q221 and yields a fragment. Depletion of *INTS10* enhanced CVB3 replication and blocked snRNA processing. Overexpression of *U1* snRNA inhibited CVB3 infection, whereas its knockdown conversely enhanced it. Similarly, knockdown of *U2* snRNA was found to promote CVB3 replication. Taken together, the 3C^pro^-mediated cleavage of INTS10 disrupts *U snRNA* processing, which in turn counteracts the inhibitory effect of snRNA *U1* and *U2* on virus replication and subverts host defenses.

## 1. Introduction

CVB, a member of the *Picornaviridae* family, is a positive-sense single-stranded RNA virus that causes acute myocarditis and pancreatitis in humans [1,2,3,4]. The elusive pathogenic mechanisms of CVB impede the development of effective interventions and preventive measures. Therefore, understanding these mechanisms is of paramount importance.

The CVB genome is approximately 7.4 kb in length, with a single open reading frame that encodes a large precursor protein [5]. This precursor protein is processed into four structural proteins (VP1–VP4) and seven nonstructural proteins (2A–2C, 3A–3D) [6,7]. The viral cysteine proteases 2A^pro^ and 3C^pro^ are involved in biological processes in both CVB and the host [8,9]. CVB 3C^pro^ cleaves MAVS and TRIF to suppress host innate immune responses [10]. 3C^pro^ inhibits RIG-I-mediated antiviral signaling by cleaving 14-3-3ε [11]. Similarly, EV-A71 promotes viral replication by cleaving the host protein double-stranded RNA-binding protein Staufen homolog 2 (Stau2) [12]. Enterovirus 3C^pro^ is transported to the nucleus via its precursor 3CD, where it cleaves transcription and regulatory factors to modulate the host’s RNA synthesis [13]. However, the precise role of CVB in this process remains unclear.

The integrator complex, comprising 12–15 subunits, regulates RNA polymerase II-transcribed RNAs and is essential for the biogenesis of small nuclear RNAs (snRNAs) and enhancer RNAs [14,15,16]. Disrupting the integrator function results in severe genetic diseases [17,18,19]. INTS10, a subunit of this complex, interacts with RNA polymerase II to mediate the 3’ end processing of *U1* and *U2* snRNAs, the core components of the spliceosome [20,21]. Overexpression of *U1* snRNA protects host cells against RNA viruses (e.g., IAV, SeV, VSV, EMCV, RSV), while its knockdown significantly increases viral burden in vitro and in vivo [22]. *U1* snRNA interacts with Tripartite motif (TRIM) 25 in the cytoplasm, promoting TRIM25-mediated RIG-I activation to enhance host defense against RNA viruses [22]. *U2* snRNA interacts with viral RNA in the cytoplasm, suppressing CVB replication [23]. Notably, INTS10 suppresses HBV replication via Interferon regulatory factor 3 (IRF3) in liver cells [24], but its relationship with CVB has not been explored.

Recently, we found that INST10 was cleaved in the cells infected with CVB, suggesting INST10 may play a role in CVB replication. This study elucidated how INST10 is cleaved in the CVB-infected cells and identified the significance of INST10 cleavage for CVB infection. Our study seeks to uncover a novel mechanism by which CVB hijacks a central component of the host’s RNA processing machinery and adds novel insights into viral pathogenesis for developing host-directed antiviral therapeutics.

## 2. Results

### 2.1. CVB3 Infection Induces the Cleavage of INTS10

**Figure 1:**
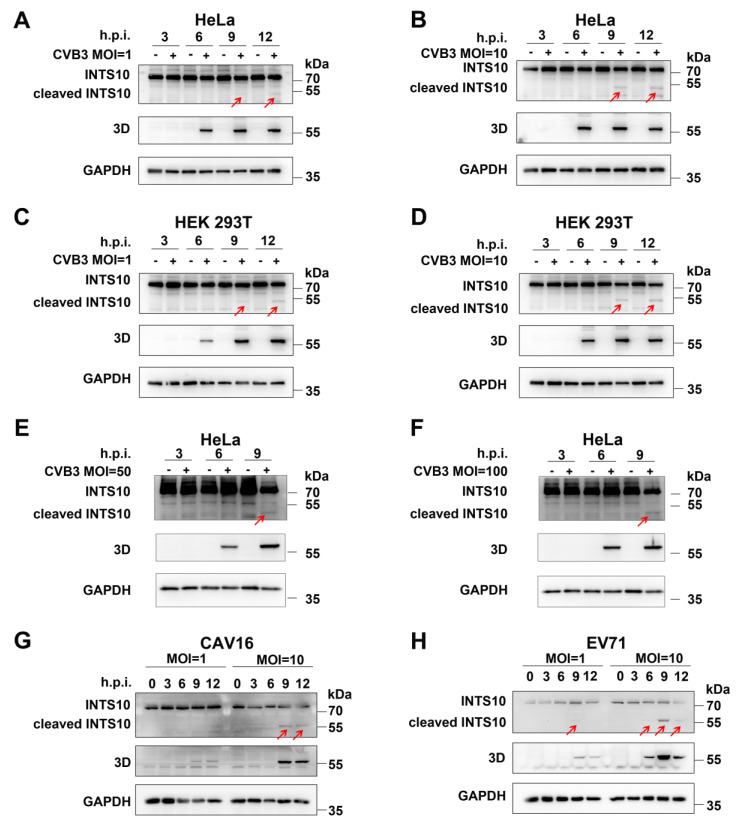
INTS10 is cleaved by CVB3, EV-A71, and CAV16. (**A**,**B**) HeLa cells were infected with CVB3 (MOI = 1 or 10). INTS10 expression was determined by Western blotting. (**C**,**D**) HEK 293T cells were infected with CVB3 (MOI = 1 or 10). INTS10 expression was determined by Western blotting. (**E**,**F**) HeLa cells were infected with CVB3 (MOI = 50 or 100). INTS10 expression was determined by Western blotting. (**G**,**H**) HeLa cells were infected with EV-A71 (**G**) and CAV16 (**H**). Red arrows indicate INTS10 cleavage fragments.

To investigate the impact of CVB3 infection on INTS10, we analyzed its expression in HeLa and HEK 293T cells infected with CVB3 (MOI = 1 or 10). At different time points post-infection, cell lysates were subjected to Western blotting analysis. We detected the effects of CVB3 infection on the expression of endogenous INTS10. The full-length INTS10 was observed with an apparent molecular mass of ~82 kDa in uninfected controls, while a truncated form of INTS10 (less than 55 kDa, red arrows) was detected in CVB3-infected HeLa and HEK 293T cells (Figure 1A–D), suggesting that INTS10 undergoes cleavage during CVB3 replication. To determine whether INTS10 cleavage is dependent on CVB3 concentration, HeLa cells were infected with CVB3 at MOI = 50 and 100 for 3, 6, and 9 h. We observed that the quality of full-length INTS10 reduced, and a small fragment with a molecular weight (MW) less than 55 kDa emerged at 9 h post-infection (Figure 1E,F, red arrows). Consistent with this observation, cleavage bands of INTS10 were also evident at 9 h post-infection with CVB3 at MOI = 50 and MOI = 100 in HEK 293T cells, as shown in Supplementary Figure S1.

To determine whether this cleavage is specific to CVB3 or whether it represents a common mechanism utilized by enteroviruses, we assessed the status of INTS10 in HeLa cells infected with EV-A71 or CAV16. Similarly to that observed in CVB3-infected cells, a cleavage fragment of INTS10 appeared in both EV-A71- and CAV16-infected cells (Figure 1G,H, red arrows), indicating that INTS10 cleavage is a shared feature of enterovirus infections.

### 2.2. CVB3 3C^pro^ Is Responsible for the Cleavage of INTS10

**Figure 2:**
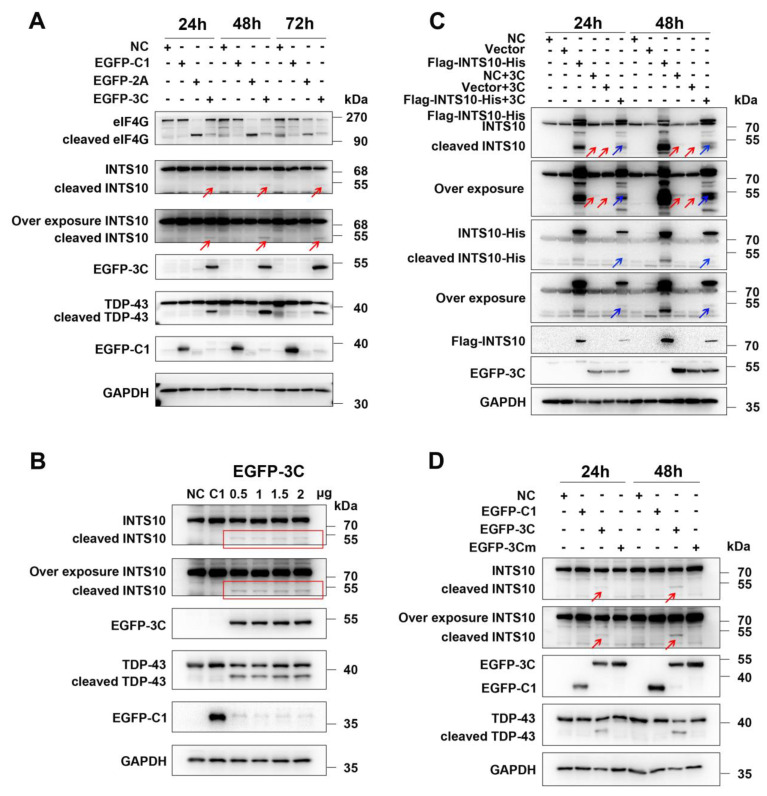
The 3C^pro^ of CVB3 induces INTS10 cleavage. (**A**) HEK 293T cells were transfected with the constructs expressing the protease 2A or 3C. INTS10 cleavage was detected by Western blotting. (**B**) HEK 293T cells were transfected with different concentrations of 3C-expressing vectors for 24 h, and INTS10 cleavage was analyzed by Western blotting. (**C**) HEK 293T cells were cotransfected with Flag-INTS10-His and 3C-expressing vectors. INTS10 cleavage was detected by Western blotting. (**D**) HEK 293T cells were transfected with constructs expressing wild-type or mutant 3C. INTS10 cleavage was detected by Western blotting. Red arrows and red frame indicate endogenous INTS10 cleavage fragments, and blue arrows indicate exogenous INTS10 cleavage fragments.

To clarify the precise mechanism by which INTS10 is cleaved, we first investigated whether CVB3-encoded viral proteases 2A^pro^ and 3C^pro^ participate in this process. Plasmids encoding CVB3 2A^pro^ and 3C^pro^ (GFP-2A and GFP-3C) were transfected into HEK 293T cells. The results showed that a cleavage fragment was only detected in cells expressing CVB3 3C^pro^, but not in GFP control or CVB3 2A^pro^-expressing cells, indicating that CVB3 3C^pro^ was involved in the cleavage of INTS10 (Figure 2A, red arrows). We also observed that INTS10 cleavage occurred when co-transfected with different amounts of 3C (Figure 2B, red frame). To further investigate whether CVB3 3C^pro^ targets INTS10, HEK 293T cells were transfected with GFP-3C plasmid, along with a plasmid expressing INTS10, with a Flag and HA tag fused at its N- and C-termini, respectively. At 48 h after transfection, cell lysates were subjected to detect 3C^pro^-mediated cleavage of INTS10 by Western blotting. We found that protein bands (~ 55 kDa of a C-terminal fragment detected by the anti-His antibody) appeared when EGFP-3C was co-transfected with INTS10 but not with the vector plasmid alone (Figure 2C, blue arrows). 

To investigate whether the proteolytic activity of 3C^pro^ is involved in INTS10 cleavage, we generated a construct expressing 3C enzyme-defective mutant (C147S). The constructs of wild-type (EGFP-3C) and mutant 3C (EGFP-3Cmut) were co-transfected into HEK 293T cells. It is obvious that the 3C enzyme-defective mutant failed to induce INTS10 cleavage compared with the wild-type 3C (Figure 2D, red arrows). These results confirmed that CVB3 3C^pro^ cleaves INTS10 protein through its proteolytic activity.

### 2.3. The CVB3 3C^pro^ Cleaves the INTS10 Protein at Residue Q221

**Figure 3:**
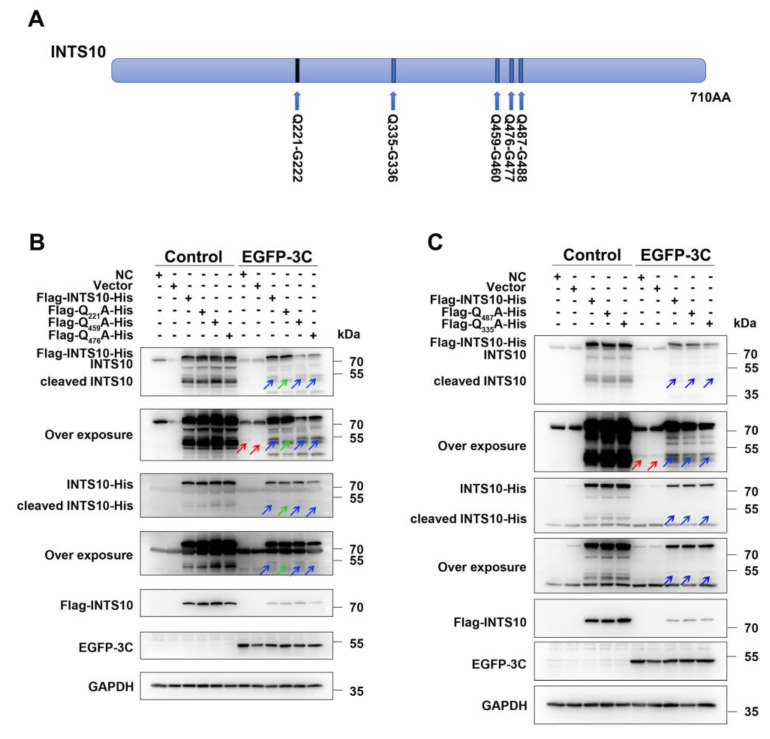
CVB3 3C^pro^ cleaves INTS10 at Q221-G222. (**A**) Schematic diagram of the INTS10 cleavage site. (**B**,**C**) HEK 293T cells were co-transfected with Flag-INTS10-His wild-type or mutant variants (Q221A, Q335A, Q459A, Q476A, and Q487A) and 3C-expressing vectors for 48 h. INTS10 cleavage was analyzed by Western blotting. Red arrows indicate endogenous INTS10 cleavage fragments. Blue arrows indicate exogenous INTS10 cleavage fragments. Green arrows indicate the absence of the INTS10 cleavage fragment at its expected migration position.

To identify the cleavage site within INTS10, we conducted mutational analysis. Certain glutamine (Q) residues like the Q-G/Q-S motif in the INTS10 sequence are recognized by the CVB3 3C^pro^. Based on the molecular weights of the cleavage products, the Q221-G222, Q335-G336, Q459-G460, Q476-G477, and Q487-G488 pairs were identified as candidate cleavage sites (Figure 3A). We generated the five following INTS10 mutants, in which Q residues were substituted with alanine (A): Q221A, Q335A, Q459A, Q476A, and Q487A. As shown in Figure 3B,C, wild-type INTS10 was cleaved by GFP-3C, producing a ~ 55kDa C-terminal fragment (Figure 3B, lane 9, blue arrows). Similarly, the Q335A, Q459A, Q476A, and Q487A mutants were also cleaved by GFP-3C (Figure 3B, lanes 11 and 12; Figure 3C, lanes 8, 9, and 10, blue arrows). In contrast, no cleavage products were detectable in the Q221A mutant co-expressed with 3C (Figure 3B, lane 10, green arrows) with anti-INTS10 or anti-His antibodies. These results demonstrate that the Q221A mutant is resistant to proteolytic cleavage, confirming that CVB3 3C^pro^ cleaves INTS10 at the Q221-G222 site.

### 2.4. Silencing Endogenous INTS10 Expression Enhances CVB3 Replication

**Figure 4:**
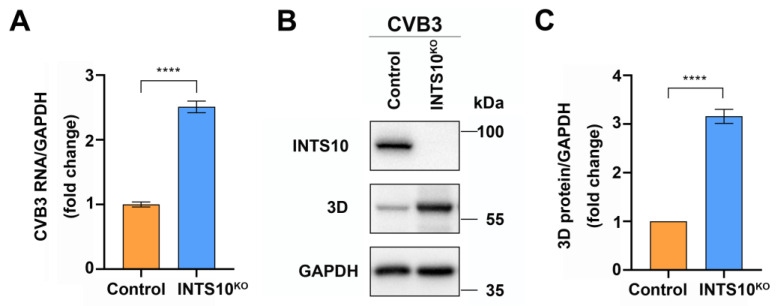
Knockout of *INTS10* enhances CVB3 replication. *INTS10*-KO HEK 293T cells and control cells were infected with CVB3 (MOI = 0.1) for 24 h. (**A**) The level of CVB3 RNA was determined by RT-qPCR. (**B**) The expression of CVB3 3D protein was determined via Western blotting. (**C**) Gray-scale densitometry of CVB3 3D protein bands. Error bars represent the standard deviation (SD). *n* = 3. **** *p* < 0.0001.

To determine whether INTS10 regulates CVB3 infection, we infected *INTS10*-KO HEK 293T cells with CVB3. Total RNA and proteins were extracted and analyzed via RT-qPCR and Western blotting. We observed that CVB3 RNA levels increased in the *INTS10*-silenced cells compared to that of control cells (Figure 4A). Similarly, viral protein 3D levels were elevated in *INTS10*-KO cells (Figure 4B,C). These results demonstrate that INTS10 exerts an inhibitory effect on CVB3 replication.

### 2.5. 3C^pro^-Mediated INTS10 Cleavage Impairs snRNA Processing and Host Defense

**Figure 5:**
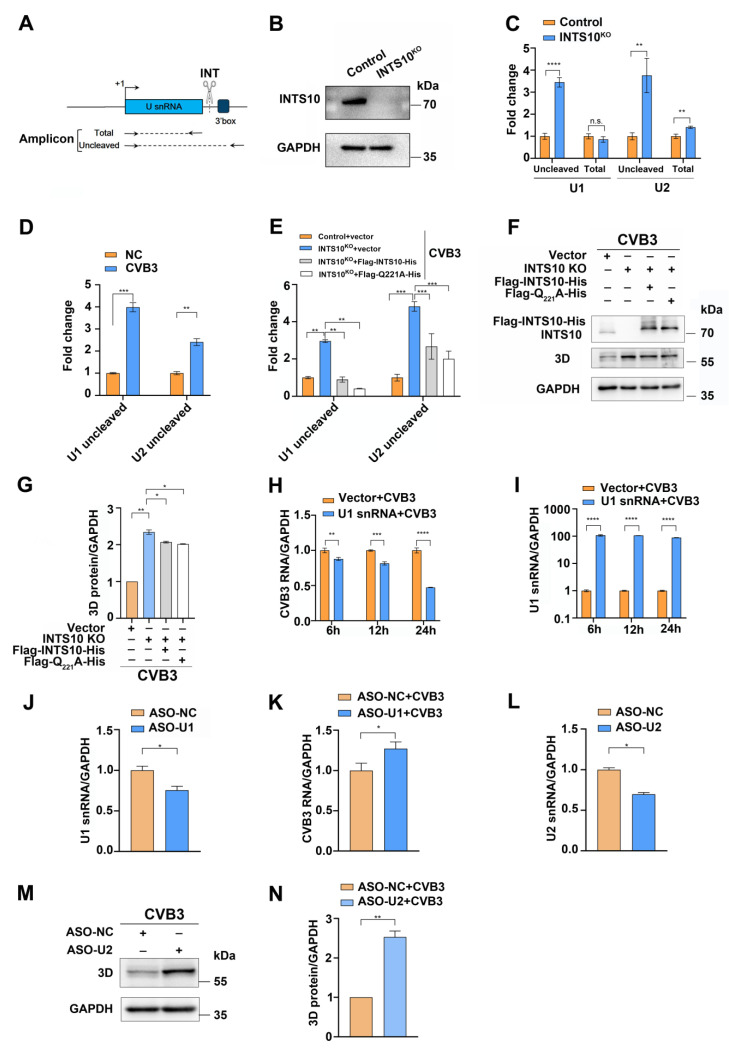
CVB3-mediated INTS10 cleavage impairs snRNA processing. (**A**) The schematic diagram of the human *U snRNA* gene showing the positions of the primers used in RT-qPCR to amplify total and uncleaved *U snRNA*. (**B**,**C**) *U1* and *U2* snRNA levels in *INTS10*-KO HEK 293T cells were determined by RT-qPCR. (**D**) HEK 293T cells were infected with CVB3 (MOI = 0.1) for 24 h. Unprocessed *U1* and *U2* snRNA levels were subjected to RT-qPCR analysis. (**E**–**G**) *INTS10*-KO HEK 293T cells transfected with Flag-INTS10-His (wild-type or Q221A mutant) were infected with CVB3 (MOI = 0.1). (**E**) Unprocessed *U1* and *U2* snRNA levels were determined by RT-qPCR. (**F**,**G**) The level of CVB3 3D protein was assessed by Western blotting. (**H**,**I**) HEK 293T cells transfected with EGFP-U1 snRNA were infected with CVB3 (MOI = 0.1) for 6, 12, and 24 h. The level of CVB3 RNA and *U1* snRNA was determined by RT-qPCR. (**J**,**K**) HEK 293T cells transfected with ASO-*U1* were infected with CVB3 at MOI of 0.1 for 24 h. The level of *U1* snRNA and CVB3 RNA was analyzed by RT-qPCR. (**L**–**N**) HEK 293T cells transfected with ASO-*U2* were infected with CVB3 (MOI = 0.1) for 24 h. (**L**) The level of *U2* snRNA was determined by RT-qPCR. (**M**,**N**) The expression of CVB3 3D protein was detected by Western blotting. Error bars: SD, *n* = 3. n.s. no significance, * *p* < 0.05, ** *p* < 0.01, *** *p* < 0.001, **** *p* < 0.0001.

INTS10, a subunit of the integrator complex, plays a critical role in the 3′-end processing of *U1* and *U2* snRNAs [14]. We hypothesized that 3C^pro^-mediated cleavage of INTS10 disrupts snRNA maturation. To test this, total and unprocessed 3′-box regions of *U1* and *U2* snRNAs were determined by RT-qPCR in *INTS10*-KO HEK 293T cells (Figure 5A). Notably, unprocessed *U1* and *U2* snRNA levels increased significantly following *INTS10* knockout (Figure 5B,C).

To determine the effect of CVB3 infection on snRNA maturation, HEK 293T cells were infected with CVB3, which resulted in elevated levels of unprocessed *U1* and *U2* snRNA (Figure 5D). To further assess the impact of CVB3 infection on snRNA maturation, *INTS10*-KO HEK 293T cells were transfected with wild-type or cleavage-resistant Q221A mutant INTS10 plasmids prior to CVB3 infection. Unprocessed *U1* and *U2* snRNA levels increased in the CVB3-infected *INTS10*-KO cells compared to the control cells. However, overexpression of either wild-type or mutant INTS10 reduced unprocessed snRNA levels compared to that of CVB3-infected cells transfected with an empty vector (Figure 5E). These observations demonstrate that INTS10 cleavage disrupts snRNA processing, leading to an accumulation of unprocessed transcripts. Moreover, both the overexpression of wild-type and mutant INTS10 inhibited the expression of the CVB3 3D protein (Figure 5F,G).

Accumulated unprocessed snRNAs correlated with reduced mature *U1* and *U2* snRNA levels. Given *U1* and *U2* snRNA’s role in restricting RNA viruses [22,23], we investigated their impact on CVB3 infection. Overexpression of *U1* snRNA significantly reduced CVB3 RNA levels (Figure 5H,I), and its knockdown elevated CVB3 RNA levels (Figure 5J,K). This indicates that *U1* snRNA inhibits CVB3 infection. Moreover, silencing *U2* snRNA significantly increased CVB3 3D protein expression (Figure 5L–N), indicating their antiviral activity. We propose that CVB3 3C^pro^ cleaves INTS10 to evade *U1* and *U2* snRNA-dependent host defenses.

## 3. Discussion

CVB3-encoded 3C^pro^ targets and disrupts host physiological activities by cleaving cellular proteins that are engaged in gene expression [9,25]. Host proteins with the consensus sequence of AXXQ/GPXX (X denotes any amino acid) could be cleaved between Q and G residues by 3C^pro^ [26,27]. In this study, we identified that INTS10, a subunit of the integrator complex, is cleaved at Q221-G222 by CVB3 3C^pro^. Silencing the expression of INTS10 promoted CVB3 replication, highlighting its critical role in restricting CVB3 infection. Furthermore, silencing the expression of *INTS10* induced the accumulation of unprocessed *U1* and *U2* snRNA, while INTS10 overexpression alleviated this accumulation. We also demonstrated that up-regulating the *U1* snRNA inhibits CVB3 replication, while silencing the *U1* snRNA promotes CVB3 replication, suggesting that *U1* snRNA acts as an intrinsic cellular restriction factor against CVB3 infection. Furthermore, the depletion of *U2* snRNA also resulted in enhanced viral replication, suggesting that *U2* snRNA also functions as an important host restriction factor for CVB3 infection. We propose that the targeted cleavage of INTS10 by CVB3 3C^pro^ serves to disrupt the cellular antiviral responses triggered during infection.

Enteroviruses have evolved diverse strategies to disrupt host cell transcription. For example, enterovirus-encoded 3C^pro^ terminates host transcription during infection [28]. Poliovirus (PV) 2A^pro^ alters pre-mRNA splicing by regulating protein shuttling between the nucleus and the cytoplasm [29]. 3D Polymerase (3D^pol^) impairs pre-mRNA splicing by disrupting pre-mRNA processing factor 8 (Prp8), reducing mRNA levels [30]. INTS10, a subunit of the integrator complex, interacts with RNA polymerase II to mediate the 3′ end processing of *U1* and *U2* snRNAs—core spliceosome components critical for transcription [20,21]. We identified that a lack of *INTS10* obstructs *U1* and *U2* snRNA processing. Moreover, *U1* snRNA is essential for host defense against multiple RNA virus infections [22], while the U2 small nuclear ribonucleoprotein (snRNP) suppresses CVB3 replication by exploiting *U2* snRNA to interact with viral genomes [23,31]. Here, our results also confirmed that *U1* and *U2* snRNA have restrictive activity to CVB3 replication, thus providing a mechanistic explanation for how INTS10 cleavage by 3C^pro^ strategically blocks host transcription to facilitate CVB3 infection.

Previous studies link INTS10 to HBV suppression via IRF3-dependent pathways, which correlates with the persistence of HBV infection [24]. Enteroviruses have developed various countermeasures against the host interferon response [32,33]. In this study, we demonstrated that silencing *INTS10* enhances CVB3 RNA and viral proteins, underscoring its role in antiviral defense. Whether INTS10’s inhibition on CVB3 is related to IFN signaling remains to be investigated.

**Figure 6:**
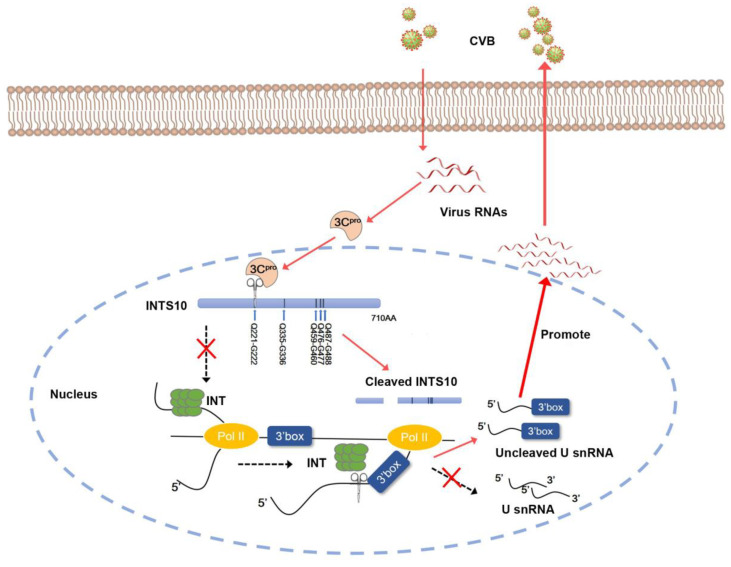
3C^pro^-mediated cleavage of INTS10 increases the *U1* and *U2* snRNA misprocessing to promote CVB3 replication.

We demonstrated that *U1* and *U2* snRNA regulate CVB3 replication by reducing levels (MOI = 0.1, 24 h) (Figure 5). Under these conditions, endogenous INTS10 is still present and subject to cleavage, leading to aberrant *U1* and *U2* snRNA processing and enhanced viral replication. Thus, it is likely that the accumulating misprocessing of *U1* and *U2* snRNA after multiple infection cycles leads to increased CVB3 replication (Figure 6). A key detail in this schematic diagram that requires further investigation is the subcellular localization of CVB3 3C^pro^-mediated cleavage of INTS10. Specifically, it remains unclear whether 3C^pro^ enters the nucleus to cleave INTS10 or whether 2A^pro^ promotes the nuclear export of INTS10, enabling its cleavage by 3C^pro^ in the cytoplasm. Previous studies have shown that in PV-infected cells, 3C^pro^ can localize to the nucleus, where it cleaves cellular transcription factors to suppress host RNA synthesis [13]. Furthermore, enterovirus 2A^pro^ can alter nuclear pore permeability and promote the nuclear export of proteins such as TDP-43, which can be cleaved by 3C^pro^ [34,35,36]. Based on these findings, we performed cytoplasmic and nuclear fractionation experiments, which revealed that during CVB infection, 3C^pro^ is present in both the cytoplasm and the nucleus (see Supplementary Figure S2). This observation supports the hypothesis that during CVB3 infection, 3C^pro^ may enter the nucleus to cleave INTS10. However, this conclusion still requires further experimental validation.

The limitation of this study is that we did not explore the role of cleaved INTS10 fragments in virus infection. Previous studies showed that the proteolytic products of the cellular proteins might interfere with the functions of their normal counterparts [11,37]. Additionally, the molecular mechanism by which *U1* snRNA exerts its inhibitory effect on CVB3 replication remains to be studied. It was reported that *U1* snRNA interacts with TRIM25 to activate RIG-I and IFN-I signaling, suggesting its role in multiple RNA viruses [22]. Future studies are worth conducting to elucidate the role of *U1* snRNA in CVB3 infection.

In summary, our findings reveal how CVB3 overcomes the restriction of snRNA on virus replication by cleaving INTS10. The cleavage of INTS10 leads to disrupted host transcription and innate immune responses, which in turn promote virus infection (Figure 6). Thus, enhancing INTS10 resistance to 3C^pro^-mediated cleavage or upregulating *U1* and *U2* snRNA could offer effective antiviral strategies.

## 4. Materials and Methods

### 4.1. Cells and Viruses

HEK 293T and HeLa cells were cultured in Dulbecco’s Modified Eagle Medium (DMEM, Gibco, Shanghai, China) supplemented with 10% fetal bovine serum (FBS, Biological Industries, Israel), and 1% penicillin/streptomycin at 37 °C. CVB3 woodruff was kindly provided by the Scrips Institute (San Diego, CA, USA). CVB3 stocks were propagated and titered in HeLa cells.

### 4.2. Plasmids and DNA Transfections

Empty vector pcDNA3.4(+)-3 × Flag-MCS-6 × His and wild-type plasmid-expressing INTS10 named pcDNA3.4(+)-3 × Flag-INTS10-6 × His were purchased from Miaoling Biology (Wuhan, China). The expression plasmids for CVB3 2A, and 3C with a GFP tag in pEGFP-C1 were generated as described previously. The CVB3 3C (C147S) and Flag-INTS10-His mutants (Q221A, Q335A, Q459A, Q476A, and Q487A) were constructed by site-specific mutagenesis. The plasmid-expressing U1 snRNA, designated as pEGFP-U1 snRNA, was constructed based on pcDNA3.1-EGFP. The following primers were used:

For Q221A:

- Forward primer: 5′-TGGGCGCCTGCATGCTGATTTTC-3′
- Reverse primer: 5′-AATCAGCATGCAGGCGCCCAGG-3′

For Q335A:

- Forward primer: 5′-GGACCTGCGAAGAGAGATGGCTGTATGC-3′
- Reverse primer: 5′-TCTCTTCGCAGGTCCTAATGCCCCG-3′

For Q459A:

- Forward primer: 5′-CGCCTTTTTATATTGACCCGCATAGATGATC-3′
- Reverse primer: 5′-ATATGATCATCTATGCGGGTCAATATAAAAAGGC-3′

For Q476A:

- Forward primer: 5′-AAATGGATCCCGCGAGAGCTGCTAAG-3′
- Reverse primer: 5′-AGCAGCTCTCGCGGGATCCATTTCT-3′

For Q487A:

- Forward primer: 5′-CAGGGTCCCCGCCCCTGTGATC-3′
- Reverse primer: 5′-ACAGGGGCGGGGACCCTGGAG-3′

For pEGFP-U1 snRNA

- Forward primer: 5′-GCGCGGAAGCTTTGATTTCATACTTACCT-3′
- Reverse primer: 5′-TATATAGAATTCCAGGGGAAAGCGCGAAC-3′

All DNA transfections were performed using Lipofectamine 2000 according to the manufacturer’s protocol (Themo Fisher Scientific, Waltham, MA, USA). Briefly, cells were seeded 24 h before transfection to allow for adherence. Transfection took place in antibiotic-free DMEM supplemented with 10% FBS. All transfections were conducted for 24 h prior to experimentation.

### 4.3. ASO Transfection

HEK 293T cells were seeded in a 12-well plate for 24 h. Cells were transfected with ASOs (GenePharma, Suzhou, China) at a final concentration of 20 nM, using siRNA-mate plus transfection reagent according to the manufacturer’s instructions (GenePharma, Suzhou, China) and incubated at 37 °C with 5% CO_2_ for 48 h. *U1* snRNA-ASO: mG*mA*mG*mA*mT*A*C*C*A*T*G*A*T*C*mA*mC*mG*mA*mA; *U2* snRNA-ASO: mA*mG*mA*mA*mC*A*G*A*T*A*C*T*A*C*A*mC*mT*mT*mG*mA.

### 4.4. Virus Infections

CVB3 viruses, EV-A71, and CAV16 were incubated with cells at the indicated MOI for 1 h in DMEM + 1% penicillin/streptomycin at 37 °C. After adsorption, the media was replaced with complete DMEM (1× DMEM, 10% FBS, 1% penicillin/streptomycin) and incubated for the designated time.

### 4.5. Western Blotting

Cells were harvested using RIPA lysis buffer (Thermo Fisher Scientific, Waltham, MA, USA) containing a protease inhibitor cocktail and 1% phenylmethylsulfonyl fluoride (PMSF, Beyotime, Shanghai, China) and were lysed for 30 min. Equal amounts of protein were separated on 10% or 12.5% SDS-PAGE gel and subsequently transferred onto a polyvinylidene difluoride (PVDF, Millipore, Billerica, MA, USA) membrane. PVDF membranes were blocked with skimmed milk for 1 h and incubated with the primary antibody overnight at 4 °C. The primary antibodies used were as follows: 1:2000 INTS10 (Proteintech, Wuhan, China); 1:1000 eIF4G (Proteintech, Wuhan, China); 1:5000 TDP-43 (Proteintech, Wuhan, China); 1:3000 GFP (ABclonal, Wuhan, China); 1:25,000 Flag (Proteintech, Wuhan, China); 1:10,000 His (Proteintech, Wuhan, China); and 1:20,000 GAPDH (ABclonal, Wuhan, China). Membranes were washed with TBST and incubated with anti-rabbit or anti-mouse IgG for 1 h at room temperature. Finally, the membrane was imaged with a FluorChem M CCD camera (ProteinSimple, Santa Clara, CA, USA) and analyzed by ImageJ software, version 1.54g.

### 4.6. Reverse Transcription Quantitative PCR (RT-qPCR)

RT-qPCR was performed using total cellular cDNA. Briefly, cells were harvested in 1 mL Trizol (Yeasen, Shanghai, China), and total cellular RNA was isolated according to the manufacturer’s protocol. A total of 1 μg of total RNA was used as a template with PrimeScript RT Enzyme Mix I (TaKaRa, Dalian, China) in a reverse transcription system. RT-qPCR was performed on a LightCycler 96 (Roche, Basel, Switzerland) using SYBR Premix Ex Taq II (TaKaRa, Dalian, China) mixed with 1 μL of the synthesized cDNA and sense/antisense primers to reach a final volume of 20 μL. Relative RNA quantity was calculated using the 2^−ΔΔCT^ method and normalized to the quantity of *GAPDH*. The following primers were used for analysis:

For *GAPDH*:

- Forward primer: 5′-CTGGGCTACACTGAGCACC-3′
- Reverse primer: 5′-AAGTGGTCGTTGAGGGCAATG-3′

For CVB3:

- Forward primer: 5′-GCACACACCCTCAAACCAGA-3′
- Reverse primer: 5′-ATGAAACACGGACACCCAAAG-3′

For *INTS10*:

- Forward primer: 5′-GCACACACCCTCAAACCAGA-3′
- Reverse primer: 5′-ATGAAACACGGACACCCAAAG-3′

For uncleaved *U1*:

- Forward primer: 5′-TACCTGGCAGGGGAGATACC-3′
- Reverse primer: 5′-GCGTACGGTCTGTTTTTGAAACTC-3′

For total *U1*:

- Forward primer: 5′-ATACCATGATCACGAAGGTGGTT-3′
- Reverse primer: 5′-CAGTCCCCCACTACCACAAATTA-3′

For uncleaved *U2*:

- Forward primer: 5’-AACATAGGTACACGTGTGCCACGG-3’
- Reverse primer: 5’-ACAAATAGCCAACGCATGCGGGGC-3’

For total *U2*:

- Forward primer: 5’-CTTCTCGGCCTTTTGGCTAAGAT-3’
- Reverse primer: 5’-GTACTGCAATACCAGGTCGATGC-3’

For *U1* snRNA:

- Forward primer: 5’-CTTACCTGGCAGGGGAGATA-3’
- Reverse primer: 5’-GCAGTCGAGTTTCCCACATT-3’

For *U2* snRNA:

- Forward primer: 5’-CTTCTCGGCCTTTTGGCTAAGAT-3’
- Reverse primer: 5’-GTACTGCAATACCAGGTCGATGC-3’

### 4.7. Statistical Analyses

Statistical data were analyzed using Graphpad Prism 8. Quantitative data were expressed as mean ± SD. Differences among test groups were analyzed by an analysis of variance (ANOVA) test or Student’s *t*-test. A value of *p* < 0.05 was considered statistically significant. All experiments were repeated three times.

## Data Availability

The original contributions presented in this study are included in the article/Supplementary Material. Further inquiries can be directed to the corresponding authors.

## Supplementary Materials

The following supporting information can be downloaded at https://www.mdpi.com/article/10.3390/ijms27020996/s1.

## Abbreviations

The following abbreviations are used in this manuscript:

INST10	Integrator subunit 10
CVB	Coxsackievirus B
3C^pro^	3C protease
2A^pro^	2A protease
Stau2	Staufen homolog 2
snRNAs	Small nuclear RNAs
TRIM	Tripartite motif
IRF3	Interferon regulatory factor 3
MW	Molecular weight
PV	Poliovirus
3D^pol^	3D Polymerase
Prp8	Pre-mRNA processing factor 8
snRNP	Small nuclear ribonucleoprotein

## References

[1] Bouin A., Gretteau P.A., Wehbe M., Renois F., N’Guyen Y., Lévêque N., Vu M.N., Tracy S., Chapman N.M., Bruneval P. (2019). Enterovirus Persistence in Cardiac Cells of Patients with Idiopathic Dilated Cardiomyopathy Is Linked to 5’ Terminal Genomic RNA-Deleted Viral Populations with Viral-Encoded Proteinase Activities. Circulation.

[2] Garmaroudi F.S., Marchant D., Hendry R., Luo H., Yang D., Ye X., Shi J., Mcmanus B.M. (2015). Coxsackievirus B3 replication and pathogenesis. Future Microbiol..

[3] Yajima T. (2011). Viral myocarditis: Potential defense mechanisms within the cardiomyocyte against virus infection. Future Microbiol..

[4] Knowlton K.U. (2008). CVB infection and mechanisms of viral cardiomyopathy. Curr. Top. Microbiol. Immunol..

[5] Fairweather D., Stafford K.A., Sung Y.K. (2012). Update on coxsackievirus B3 myocarditis. Curr. Opin. Rheumatol..

[6] Kemball C.C., Alirezaei M., Whitton J.L. (2010). Type B coxsackieviruses and their interactions with the innate and adaptive immune systems. Future Microbiol..

[7] Tong L., Lin L., Zhao W., Wang B., Wu S., Liu H., Zhong X., Cui Y., Gu H., Zhang F. (2011). Destabilization of coxsackievirus b3 genome integrated with enhanced green fluorescent protein gene. Intervirology.

[8] Hanson P.J., Hossain A.R., Qiu Y., Zhang H.M., Zhao G., Li C., Lin V., Sulaimon S., Vlok M., Fung G. (2019). Cleavage and Sub-Cellular Redistribution of Nuclear Pore Protein 98 by Coxsackievirus B3 Protease 2A Impairs Cardioprotection. Front. Cell. Infect. Microbiol..

[9] Lei X., Han N., Xiao X., Jin Q., He B., Wang J. (2014). Enterovirus 71 3C Inhibits Cytokine Expression through Cleavage of the TAK1/TAB1/TAB2/TAB3 Complex. J. Virol..

[10] Mukherjee A., Morosky S.A., Delorme-Axford E., Dybdahl-Sissoko N., Oberste M.S., Wang T., Coyne C.B. (2011). The coxsackievirus B 3C protease cleaves MAVS and TRIF to attenuate host type I interferon and apoptotic signaling. PLoS Pathog..

[11] Andrews D.D.T., Vlok M., Akbari Bani D., Hay B.N., Mohamud Y., Foster L.J., Luo H., Overall C.M., Jan E. (2023). Cleavage of 14-3-3ε by the enteroviral 3C protease dampens RIG-I-mediated antiviral signaling. J. Virol..

[12] Li H., Song J., Deng Z., Yao Y., Qiao W., Tan J. (2024). Cleavage of Stau2 by 3C protease promotes EV-A71 replication. Virol. J..

[13] De Jesús-González L.A., Palacios-Rápalo S., Reyes-Ruiz J.M., Osuna-Ramos J.F., Cordero-Rivera C.D., Farfan-Morales C.N., Gutiérrez-Escolano A.L., Del Ángel R.M. (2021). The Nuclear Pore Complex Is a Key Target of Viral Proteases to Promote Viral Replication. Viruses.

[14] Mendoza-Figueroa M.S., Tatomer D.C., Wilusz J.E. (2020). The Integrator Complex in Transcription and Development. Trends Biochem. Sci..

[15] Razew M., Fraudeau A., Pfleiderer M.M., Linares R., Galej W.P. (2024). Structural basis of the Integrator complex assembly and association with transcription factors. Mol. Cell.

[16] Lai F., Gardini A., Zhang A., Shiekhattar R. (2015). Integrator mediates the biogenesis of enhancer RNAs. Nature.

[17] Tepe B., Macke E.L., Niceta M., Weisz Hubshman M., Kanca O., Schultz-Rogers L., Zarate Y.A., Schaefer G.B., Granadillo De Luque J.L., Wegner D.J. (2023). Bi-allelic variants in INTS11 are associated with a complex neurological disorder. Am. J. Hum. Genet..

[18] Oegema R., Baillat D., Schot R., van Unen L.M., Brooks A., Kia S.K., Hoogeboom A.J.M., Xia Z., Li W., Cesaroni M. (2017). Human mutations in integrator complex subunits link transcriptome integrity to brain development. PLoS Genet..

[19] Tilley F.C., Arrondel C., Chhuon C., Boisson M., Cagnard N., Parisot M., Menara G., Lefort N., Guerrera I.C., Bole-Feysot C. (2021). Disruption of pathways regulated by Integrator complex in Galloway-Mowat syndrome due to WDR73 mutations. Sci. Rep..

[20] Yamamoto J., Hagiwara Y., Chiba K., Isobe T., Narita T., Handa H., Yamaguchi Y. (2014). DSIF and NELF interact with Integrator to specify the correct post-transcriptional fate of snRNA genes. Nat. Commun..

[21] Sabath K., Stäubli M.L., Marti S., Leitner A., Moes M., Jonas S. (2020). INTS10-INTS13-INTS14 form a functional module of Integrator that binds nucleic acids and the cleavage module. Nat. Commun..

[22] Zhang F., Liu S., Qiao Z., Li L., Han Y., Sun J., Ge C., Zhu J., Li D., Yao H. (2024). Housekeeping U1 snRNA facilitates antiviral innate immunity by promoting TRIM25-mediated RIG-I activation. Cell Rep..

[23] Kamel W., Ruscica V., Embarc-Buh A., Laurent Z.R.D., Garcia-Moreno M., Demyanenko Y., Orton R.J., Noerenberg M., Madhusudhan M., Iselin L. (2024). Alphavirus infection triggers selective cytoplasmic translocation of nuclear RBPs with moonlighting antiviral roles. Mol. Cell.

[24] Li Y.F., Si L.L., Zhai Y., Hu Y.L., Hu Z.B., Bei J.X., Xie B.B., Ren Q., Cao P.B., Yang F. (2016). Genome-wide association study identifies 8p21.3 associated with persistent hepatitis B virus infection among Chinese. Nat. Commun..

[25] Rui Y., Su J., Wang H., Chang J., Wang S., Zheng W., Cai Y., Wei W., Gordy J.T., Markham R. (2017). Disruption of MDA5-Mediated Innate Immune Responses by the 3C Proteins of Coxsackievirus A16, Coxsackievirus A6, and Enterovirus D68. J. Virol..

[26] Fan W., McDougal M.B., Schoggins J.W., Schultz-Cherry S. (2022). Enterovirus 3C Protease Cleaves TRIM7 To Dampen Its Antiviral Activity. J. Virol..

[27] Zhang D., Xie Y., Cao J., Huang L., Fan W., Dutch R.E. (2025). Enteroviral 3C protease cleaves N4BP1 to impair the host inflammatory response. J. Virol..

[28] Sharma R., Raychaudhuri S., Dasgupta A. (2004). Nuclear entry of poliovirus protease-polymerase precursor 3CD: Implications for host cell transcription shut-off. Virology.

[29] Álvarez E., Castelló A., Carrasco L., Izquierdo J.M. (2013). Poliovirus 2A protease triggers a selective nucleo-cytoplasmic redistribution of splicing factors to regulate alternative pre-mRNA splicing. PLoS ONE.

[30] Liu Y.C., Kuo R.L., Lin J.Y., Huang P.N., Huang Y., Liu H., Arnold J.J., Chen S.J., Wang R.Y., Cameron C.E. (2014). Cytoplasmic viral RNA-dependent RNA polymerase disrupts the intracellular splicing machinery by entering the nucleus and interfering with Prp8. PLoS Pathog..

[31] Castello A., Kamel W. (2025). Nuclear RNA-binding proteins meet cytoplasmic viruses. RNA.

[32] Liang T., Zhang Z., Bai Z., Xu L., Xu W. (2024). STAT3 Increases CVB3 Replication and Acute Pancreatitis and Myocarditis Pathology via Impeding Nuclear Translocation of STAT1 and Interferon-Stimulated Gene Expression. Int. J. Mol. Sci..

[33] Feng Q., Langereis M.A., Lork M., Nguyen M., Hato S.V., Lanke K., Emdad L., Bhoopathi P., Fisher P.B., Lloyd R.E. (2014). Enterovirus 2Apro targets MDA5 and MAVS in infected cells. J. Virol..

[34] Park N., Schweers N.J., Gustin K.E. (2015). Selective Removal of FG Repeat Domains from the Nuclear Pore Complex by Enterovirus 2A(pro). J. Virol..

[35] Wo X., Yuan Y., Xu Y., Chen Y., Wang Y., Zhao S., Lin L., Zhong X., Wang Y., Zhong Z. (2020). TAR DNA-Binding Protein 43 is Cleaved by the Protease 3C of Enterovirus A71. Virol. Sin..

[36] Zhang L., Yang J., Li H., Zhang Z., Ji Z., Zhao L., Wei W. (2023). Enterovirus D68 Infection Induces TDP-43 Cleavage, Aggregation, and Neurotoxicity. J. Virol..

[37] Li X., Guo H., Yang J., Liu X., Li H., Yang W., Zhang L., Li Y., Wei W. (2024). Enterovirus D68 3C protease antagonizes type I interferon signaling by cleaving signal transducer and activator of transcription 1. J. Virol..

