# Supplementary figures and images for: Coxsackievirus B3 Cleaves INTS10 Through 3C Protease to Facilitate Its Replication
Source: Int J Mol Sci. 2026 Jan 19;27(2):996. doi: 10.3390/ijms27020996 (PMC12841665; doi:10.3390/ijms27020996)

Supplementary Figure S1

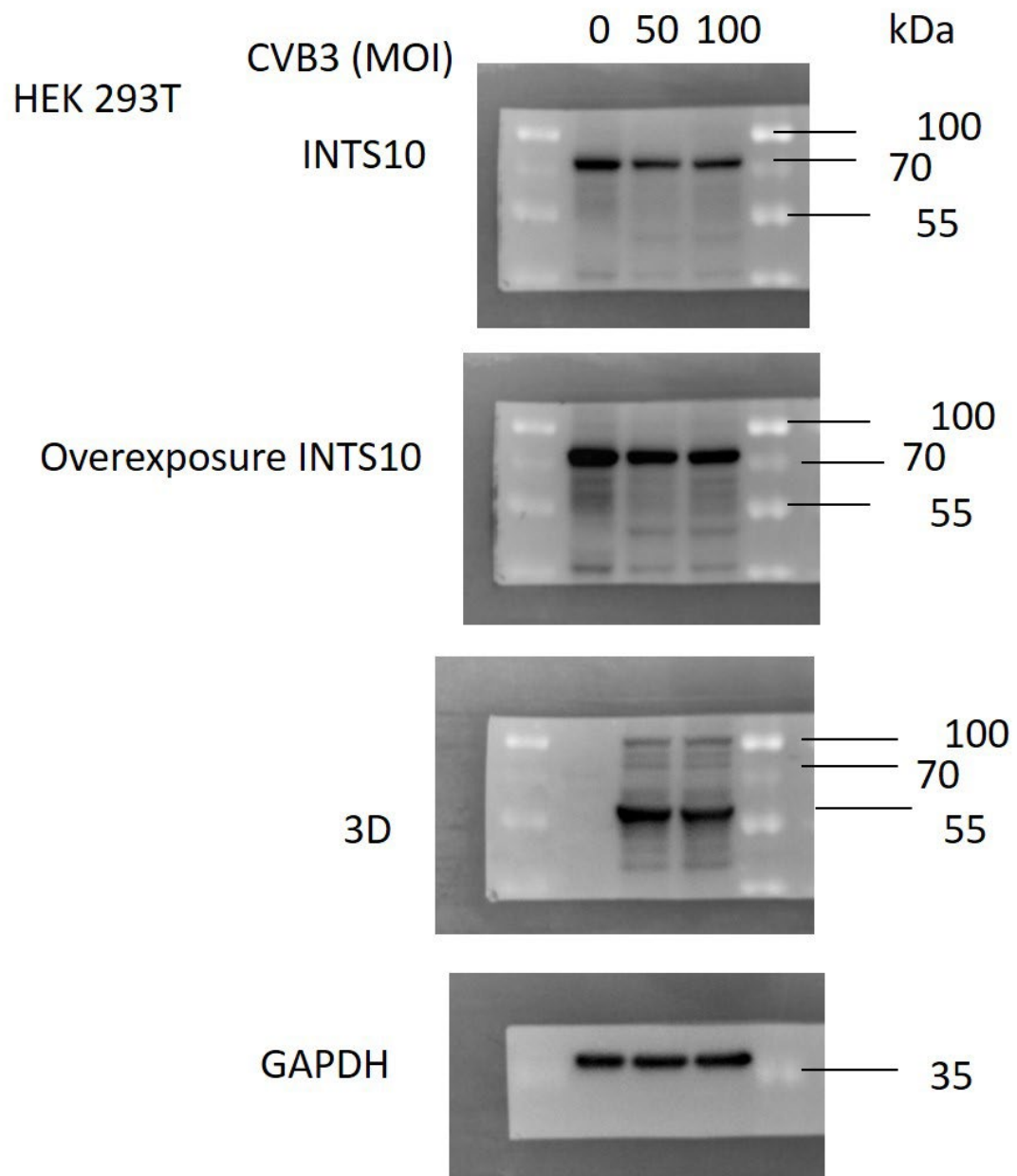

Supplementary Figure S2

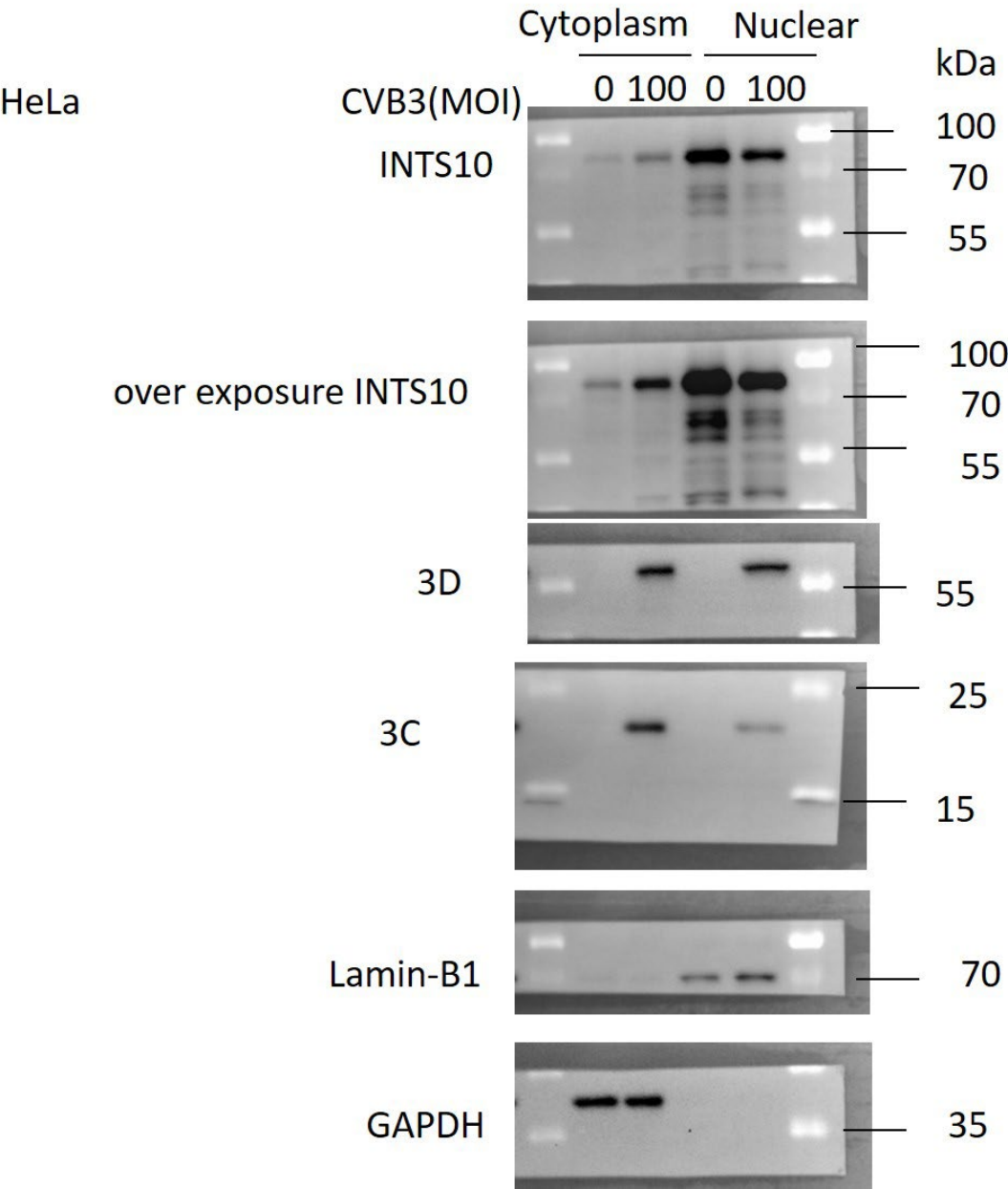

Supplement: Supplementary file 1 [file ijms-27-00996-s001.zip › Supplementary Figures.pdf]
